# Diabetes

**Published:** 1904-12-17

**Authors:** 


### PROGRESS IN MEDICINE AND SURGERY.

### DIABETES.

(*Continued from page* 193.)

Spinal Lesions.—Williamson [9] has described some degenerative processes in the spinal cord affecting the posterior columns, mainly in the cervical region. The most recent of his cases was that of a man who, after recovering from symptoms of coma, finally died of asthenia. Goll's column is mainly affected, and Burdach's only very slightly, and the condition ends abruptly when the posterior roots leave the cord. Increase of neuroglia and degeneration of nerve-fibres are observed histologically. The change is considered secondary, and due to the action of diabetic blood on that part of the axis-cylinder which is not protected by a myelin sheath. In some cases loss of knee-jerk, with no other nerve symptom, may be due not to neuritis, but to this condition.

Neuritis.—The symptoms of nervous disorder as a complication of diabetes were first described by Pavy [10] in 1885, though then attributed by him to a lesion in the spinal cord. The morbid process is now described by him as a subdued form of inflammation leading to degeneration and ultimate necrosis of the proper nerve-texture. The symptoms will depend on the particular nerve-fibrils involved and the extent of the morbid change, and may be motor, sensory, vasomotor, or nutritional. As a motor symptom cramp may occur without evidence of neuritis in the early stages, and may be the direct effect of the sugar on the muscles. The motor disturbance corresponds to that of locomotor ataxia in the staggering gait, loss of co-ordination, and marked preference for the lower limbs; the Argyll-Robertson pupil, rectal and vesical derangements, and Romberg's symptom are absent. Fibrillary contractions may occur. Sensory symptoms are often marked and may be mistaken for gout

Localised hyperæsthesia, numbness, tenderness of the feet, aching pains in the limbs, "lightning" pains and tenderness in the flesh and bones may occur. These pains are often worse at night. Pain may less commonly be felt in the arms, and sometimes "girdle-pains" occur. Absent knee-jerks are an early sign, and in chronic cases in middle or later life which have not been kept under control it is unusual for the knee-jerks to be present. Vasomotor symptoms include local flushings and pallor and local areas of sweating. Nutrition is affected partly through disorders of the vasomotor nerves and partly through loss of influence of nerve power on the nutritive processes in the tissues. Gangrene, allied to Raynaud's disease, in most cases is a result of neuritis, as is perforating sore. Trophic lesions may also result from the direct action of the sugar on the tissues—there are dermatoses such as xanthoma, boils and carbuncles. The neuritis is mostly symmetrical and usually affects the lower limbs, but it may be unilateral. Occasionally one nerve only, as the sciatic or brachial is affected. Abdominal pain and tenderness are occasionally noted, and affection of the pneumogastrics may occur, leading to severe anginal attacks and vomiting. In spite of the alarming nature of the symptoms the latter cases do well under treatment. The third nerve may be affected, ptosis and external squint resulting. Pavy strongly defends the view that the neuritis is due to the presence of sugar in the blood. He denies that the acetone bodies have any connection with neuritis, the condition is met with mainly in the slighter "alimentary" forms in which those substances are not present. Neuritis does not occur in cases well kept in hand, and is never present when sugar is absent, except in those cases where sugar has only recently been eliminated from the urine and the nerves have not had time to recover. The actual pain generally disappears a week or two after dieting has been begun. Obstinate cases occasionally occur. Conjoined with proper diet, opium or codeia, 5 grains of potassium iodide and 10 grains of potassium bromide may be given thrice daily. The galvanic current is satisfactory in obstinate cases, and for great superficial pain linimentum aconiti may be cautiously applied.

[9] Brit. Med. Jour., Jan. 16, 1904, p. 122. [10] Lancet, July 2, 1904, p. 17, and July 9, 1904, p. 71.

*(To be continued.)*

